# Influence of childhood asthma on dental caries: A longitudinal study

**DOI:** 10.1002/cre2.436

**Published:** 2021-05-08

**Authors:** Tomi Samec, Bennett Tochukwu Amaechi, Janja Jan

**Affiliations:** ^1^ Department of Dental Diseases and Endodontology, Faculty of Medicine University of Ljubljana Ljubljana Slovenia; ^2^ Department of Endodontics University Dental Clinic, University Medical Center Ljubljana Slovenia; ^3^ Department of Comprehensive Dentistry University of Texas Health San Antonio San Antonio Texas USA

**Keywords:** anti‐asthmatic medicines, asthma, dental caries, longitudinal study

## Abstract

**Objectives:**

The study investigated the influence of childhood asthma on dental caries development and caries risk factors among children with asthma in Slovenia.

**Material and methods:**

The study population consisted of 2–17 years old children (*n* = 138), who had used anti‐asthmatic medicines for at least 1 year. Controls were their non‐asthmatic siblings (*n* = 140). International Caries Detection and Assessment System‐II was used to assess caries status. After 3 years, 106 baseline participants (53 asthmatic and 53 siblings) were reexamined. Questionnaires completed by parents and data from the patients' medical records provided information on demographics, child's medical history, medication usage, and oral health behaviors. Additional 308 asthmatic children were examined to assess caries risk factors among children with asthma.

**Results:**

Asthmatic children had significantly higher mean d_12_fs and D_12_MFS (*p* ≤ 0.05), and fewer caries‐free individuals (*p* ≤ 0.01). In asthmatic children, 3 years mean increment in D_12_MFS was significantly higher (*p* ≤ 0.05). Furthermore, progression over 3 years from sound tooth surfaces to decayed cavitated and filled lesions in primary and permanent teeth were present in significantly higher (*p* ≤ 0.05) percentage, and likewise transition from decayed cavitated lesions to missing tooth surfaces because of caries in primary teeth, and from filled to filled non‐cavitated and cavitated lesions in permanent teeth. Lower caries experience in asthmatic children was associated with lower doses of inhaled glucocorticoid use, leucotriene antagonist use, and daily milk and cheese consumption.

**Conclusions:**

Asthmatic children who had used anti‐asthmatic medicines had higher caries experience and higher caries progression over 3 years in both primary and permanent dentitions. Besides anti‐asthmatic medicines, other factors were associated with higher caries experience in asthmatic children.

## 1 INTRODUCTION

Asthma is usually characterized by chronic airway inflammation, and defined by the history of respiratory symptoms such as wheezing, shortness of breath, chest tightness, and cough that vary over time and in intensity, together with variable expiratory airflow limitation (Reddel et al., 2015). It is a serious global health problem affecting all age groups, with global prevalence of up to 20% of children aged 6–7 years experiencing severe wheezing episodes within a year (Lai et al., 2009). The treatment of asthma starts with avoidance to stimuli, but controlling the symptoms with anti‐asthmatic medicines is the main component of most asthma treatments. Pharmacological management of chronic childhood asthma involves two main categories of drugs: bronchodilators and anti‐inflammatory agents. Children with mild asthma often are managed only with inhaled β2‐agonist bronchodilators. Inhaled glucocorticoids are effective anti‐inflammatory agents recommended for use in children with moderate to severe asthma. If child's asthma is not well controlled by the above medications, alternative treatments include leukotriene receptor antagonist (Castillo et al., 2017).

Dental caries is the most prevalent and ubiquitous noncommunicable disease (Meyer & Enax, 2018). It develops through a complex interaction over time between acid‐producing bacteria and fermentable carbohydrate, and many host factors including teeth and saliva (Selwitz et al., 2007). The caries process is a continuum resulting from many cycles of demineralization and remineralization, which is the body's natural attempt of repairing caries lesions. Initial demineralization is often subclinical but can lead to the development of caries lesions, which range from incipient areas of increased enamel opacity and porosity to frank cavitation. Risk of dental caries development includes physical, biological, environmental, behavioral, and lifestyle‐related factors, such as presence of dental plaque, high numbers of cariogenic bacteria, inadequate salivary flow, insufficient fluoride exposure, poor oral hygiene, inappropriate sugar consumption, and usage of various medicines (Selwitz et al., 2007).

A link between asthma and dental caries has long been discussed (Alavaikko et al., 2011) but the results seem to be conflicting. A systematic review with meta‐analysis reported that asthma doubles the risk of dental caries in both the primary and permanent dentitions (Alavaikko et al., 2011). Although the authors were unable to determine a mechanism to explain the link, they suggested use of asthma medication or a factor related to asthma per se, such as inflammation. An earlier review by Maupomé et al. (2010) claimed there is no strong evidence for a causal relation between caries and asthma. In 2019, another systematic review provided evidence that asthmatic subjects had nearly 1.5 times higher odds of the occurrence of dental caries in both dentitions (Agostini et al., 2019). The inconclusive results could be attributed to several limitations associated to methodological aspects and definitions of asthma and dental caries (Agostini et al., 2019). A need for longitudinal cohort studies is evident (Agostini et al., 2019; Alavaikko et al., 2011; Maupomé et al., 2010), to provide further knowledge to establish a causal relation between asthma and dental caries.

Potential mechanisms by which asthma could increase caries development include decreased salivary flow rate (Paganini et al., 2011; Ryberg et al., 1991), changes in saliva composition and its pH, lower plaque pH values (Stensson, Wendt, Koch, Oldaeus, et al., 2010), increased salivary levels of Streptococcus mutans (Botelho et al., 2011), and more frequent consumption of sweet drinks (Samec et al., 2013; Stensson, Wendt, Koch, Oldaeus, et al., 2010), which could be explained by frequent mouth breathing by asthmatic children leading to dry mouth and quest for drink (Samec et al., 2013; Stensson et al., 2008; Stensson, Wendt, Koch, Oldaeus, et al., 2010). The role of asthma medication is likely to be a contributing factor.

In our previous study, we showed asthmatic children had significantly higher prevalence of caries on primary and permanent teeth (Samec et al., 2013). In multivariate regression analysis, various risk factors were associated with caries experience of asthmatic children, indicating a need for further studies. The objective of the present study was therefore to examine the influence of childhood asthma on dental caries development and caries risk factors among asthmatic children in Slovenia. To assess the influence of asthma on the progression of dental caries, and as such the threat to the health of the dentition, a longitudinal design was utilized. To have a more homogenous group by disease severity, the asthmatic children must have used anti‐asthmatic medicines daily. We investigated children with asthma and healthy control siblings to infer genetic and environmental contributions. International Caries Detection and Assessment System‐II (ICDAS II) scoring criteria, which measures the disease process at its different stages, was used.

## 2 MATERIALS AND METHODS

### 2.1 Study population

The study population consisted of 2–17‐year‐old children under treatment for chronic bronchial asthma at the University Children's Hospital, Ljubljana, Slovenia. To be eligible for the study, the asthmatic children must have used anti‐asthmatic medicines daily for at least 1 year and had physician‐diagnosed asthma. Children suffering from additional diseases such as heart diseases, gastro‐esophageal reflux, chromosomal abnormalities, infectious diseases, eating disorders, and frequent vomiting were excluded from the study. Controls were non‐asthmatic siblings of the asthmatic study patients. In 2008, we examined 278 children (138 asthmatic children and 140 siblings). Baseline participants were followed up in 2011, when we reexamined 106 children (53 asthmatic children and 53 siblings). We examined whether there were systematic differences between parents and children who participated in follow‐up and those who dropped out. There was no significant difference between the two groups in terms of dental caries and demographic characteristics. For the purpose of the study, the population was divided into three age groups based on the stage of development of the dentition. The first age group was composed of 2‐ to 6‐year‐old children (*n* = 72), the second age group of 7‐ to 12‐year‐old children (*n* = 153), and the third age group of 13‐ to 17‐year‐old children (*n* = 53). The study protocol was approved by ethics committee at the Ministry of Health in Slovenia (No. 165/07/09). All the parents gave written informed consent for inclusion in this study.

In the second part of the study, we assessed the influence of various confounding factors on caries experience in the group of asthmatic children. Additional 308 asthmatic children were examined. Thus, this cohort group was composed of 446 2–17 years old asthmatic children.

Dental caries assessment was carried out at the University Dental Clinic in a dental chair under artificial light by two calibrated dentists using a standard dental mirror and rounded dental probe. The dental examiners were blinded to children with and without asthma. Children had their teeth cleaned before the examination. Caries status was determined by the number of decayed [non‐cavitated primary (d_1_) and permanent (D_1_) and cavitated primary (d_2_) and permanent (D_2_)], missing (M), and filled (f/F) surfaces in primary (dfs) and permanent (DMFS) teeth through clinical examination using the subtle ICDAS II scoring criteria (Ismail et al., 2007). The basic codes range from measurement of the first visible carious change in enamel caused by carious demineralization (code 1) to extensive cavitation with visible dentin (code 6). Radiograph was not included in the examination. Data on caries experience were presented in primary teeth as d_1_fs (non‐cavitated decayed and filled surfaces), d_2_fs (cavitated decayed and filled surfaces), d_12_fs (non‐cavitated, cavitated, and filled surfaces), and in permanent teeth as D_1_MFS (non‐cavitated decayed, missing, and filled surfaces), D_2_MFS (cavitated decayed, missing, and filled surfaces), D_12_MFS (non‐cavitated, cavitated, missing, and filled surfaces).

To measure the transition of caries in the longitudinal study, a scoring system from Ismail et al. (2011) was used. It incorporates transition of non‐cavitated lesions and reversals at the tooth surface level. Data in primary and permanent teeth were presented as s/S (sound tooth surfaces), dnc/DNC (decayed non‐cavitated lesions), dc/DC (decayed cavitated lesions), f/F (filled lesions), fnc/FNC (filled non‐cavitated lesions), fc/FC (filled cavitated lesions), m/M (missing tooth surfaces because of caries), m0/M0 (missing tooth surfaces because of other reasons than caries), x/X (not‐examined or excluded tooth surfaces).

To ensure accurate assessment for dental caries, the examiners were calibrated prior to the study by a cariologist, who is experienced in caries diagnosis. Intra‐ and inter‐examiner reproducibility was tested on 10% of the children. Weighted Cohen's kappa values for intra‐examiner reproducibility were 0.87 and 0.91 and for inter‐examiner reproducibility 0.81. These values met the 0.80 pre‐established value for qualification. Agreement of clinical assessments was therefore established to be good which validated the examination procedure.

Questionnaires completed by parents and data from the patients' medical records provided information on demographics, medical history, medication usage, dietary history, oral hygiene habits, fluoride exposure, and for asthmatic children also on type, dose, frequency, length, and mode of medicine application. For the glucocorticoid dose, we used the dose the children had been using for the previous 6 months.

### 2.2 Statistical analysis

A chi‐square test was used to test the distribution of subjects between groups for categorical independent variables. Mann–Whitney *U*‐tests were used to test the association between dental caries as dependent variable and independent variables. Children were categorized according to frequency of medicine application (1, 2 times/day), spacer use (no, yes), mouth rinsing after medicine application (no, yes), sugar content in medications (no, yes), length of medicine applications (1–3, ≥4 years), consumption of food and drinks (≤5, >5 times/day), frequency of toothbrushing (1, 2, 3 times/day), glucocorticoid dose (50, 125, 250 μg), leucotriene antagonist use (no, yes), and parents' education (elementary school, vocational school, secondary school, high school, university, postgraduate studies). The data were analyzed using the SPSS 26.0 statistical software package for Windows (SPSS Inc., Chicago, IL, USA). The level of statistical significance was set at *p* ≤ 0.05.

## 3 RESULTS

At baseline in year 2008, 278 children (average age 9.14 ± 3.56 years) were included. There was no statistically significant difference between the asthmatic children and non‐asthmatic siblings with respect to age, fluoride intake, dietary habits, oral hygiene, last dental visits, and parents' education.

The mean length of anti‐asthmatic medication use in 138 asthmatic children was 5.46 ± 3.32 years. 31.9% asthmatic children used medicines from one up to 3 years, 68.1% used them for more than 4 years. All asthmatic children used glucocorticoid daily and bronchodilatator as circumstances required, 18.8% used additional leucotriene antagonists, and 5.1% used antihistamines. Medicines in metered‐dose inhaled form were used by 75.2% asthmatic children, 24.8% used them in dry powder inhaled form. Inhalers with spacer were used by 65.9% children. After medicine application, 76.8% asthmatic children rinsed their mouths with water. Non‐sugar‐containing medications were used by 63.8% children.

Asthmatic children had significantly (*p* < 0.05) lower prevalence of sound tooth surfaces in the first and third age groups than their non‐asthmatic siblings. Significantly (*p* < 0.01) a greater number of their non‐asthmatic siblings were caries‐free in the first and third age groups when compared with the asthmatic children (Table 1).

**TABLE 1:** Prevalence of sound tooth surfaces and caries‐free children in primary and permanent teeth in different age groups in year 2008 in asthmatic children and non‐asthmatic siblings

	Age group	*N*	Sound tooth surfaces (mean ± SD)	Caries‐free children*n* (%)
Asthmatic children	2–6	72	82.17 ± 18.46**	4 (13.8%)***
	7–12	153	88.69 ± 31.29	8 (9.9.%)
	13–17	53	134.86 ± 13.67*	0 (0%)***
Non‐asthmatic siblings	2–6	72	91.79 ± 12.41**	23 (53.5%)***
	7–12	153	87.96 ± 37.76	15 (20.8%)
	13–17	53	142.60 ± 11.46*	8 (32.0%)***

* *p* < 0.05, Mann–Whitney *U*‐test.

** *p* < 0.01, Mann–Whitney *U*‐test.

*** *p* < 0.05, test *χ* ^2^.

**FIGURE 1:**
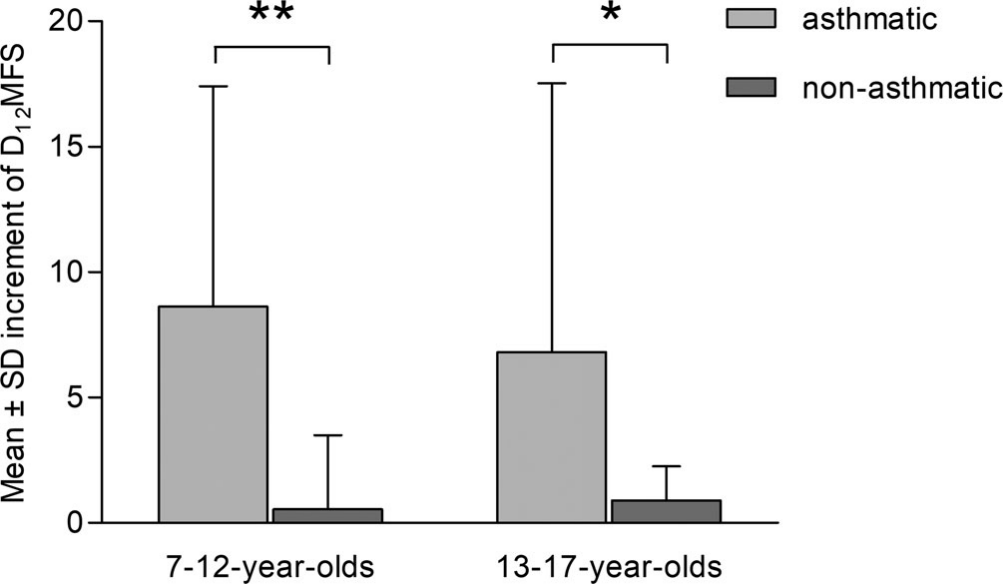
Mean ± SD increment of D12MFS (non‐cavitated, cavitated, missing, and filled surfaces) in asthmatic children and non‐asthmatic siblings. Statistically significant differences (Mann–Whitney *U*‐test) between the asthmatic children group and non‐asthmatic siblings group are marked with (*p < 0.05) and (**p < 0.01)

We focused on 106 children whose tooth surfaces data were collected in both 2008 and 2011. Asthmatic children had significantly (*p* < 0.05) higher mean d_12_fs in year 2008 and in 2011 compared to non‐asthmatic siblings on their primary teeth, as well as significantly higher mean D_12_MFS on their permanent teeth (Table 2). The 3‐year mean increment of D_12_MFS was higher among asthmatic children in the second and third age group in permanent teeth than that from non‐asthmatic siblings (Figure 1).

**TABLE 2:** Mean ± SD d_12_fs and mean ± SD D_12_MFS in year 2008 and 2011 in asthmatic children and non‐asthmatic siblings

	Age group	*N*	d_12_fs 2008 (mean ± SD)	d_12_fs 2011 (mean ± SD)	D_12_MFS 2008 (mean ± SD)	D_12_MFS 2011 (mean ± SD)
Asthmatic children	2–6	39	10.94 ± 9.71*	8.88 ± 7.71**	–	–
	7–12	51	–	–	4.24 ± 4.60	12.86 ± 10.10**
	13–17	16	–	–	12.29 ± 7.65*	19.14 ± 13.86**
Non‐asthmatic siblings	2–6	39	4.09 ± 5.46*	2.91 ± 3.88**	–	–
	7–12	51	–	–	2.32 ± 3.31	2.86 ± 3.44**
	13–17	16	–	–	4.11 ± 5.11*	5.00 ± 4.95**

* *p* < 0.05, Mann–Whitney *U*‐test.

** *p* < 0.01, Mann–Whitney *U*‐test.

Caries increment over 3 years is presented in Tables 3 and 4. Asthmatic children had significantly (*p* < 0.05) more dc on their primary teeth in year 2008 and 2011, and in year 2011 significantly more f, fc, and m (Table 3). There was a significantly higher increment of m on primary teeth among asthmatic children.

**TABLE 3:** Number and percent of s (sound tooth surfaces), dnc (decayed non‐cavitated lesions), dc (decayed cavitated lesions), f (filled lesions), fnc (filled non‐cavitated lesions), fc (filled cavitated lesions), m (missing tooth surfaces, because of caries), m0 (missing tooth surfaces, because of other reasons than caries), x (not examined or excluded tooth surfaces) in year 2008, 2011, and caries increment between year 2008 and 2011 in asthmatic children and non‐asthmatic siblings (*n* = 106) in primary teeth

	Year 2008				Year 2011				Caries increment over 3 years			
	Asthmatic children		Non‐asthmatic siblings		Asthmatic children		Non‐asthmatic siblings		Asthmatic children		Non‐asthmatic siblings	
Primary teeth	Surfaces	%	Surfaces	%	Surfaces	%	Surfaces	%	Surfaces	%	Surfaces	%
s	2148	40.53%	2615	49.34%	994	18.75%	1441	27.19%	−1154	−21.78%	−1174	−22.15%
dnc	48	0.91%	26	0.49%	6	0.11%	15	0.28%	−42	−0.80%	−11	−0.21%
dc	192*	3.62%*	68*	1.28%*	51*	0.96%*	5*	0.09%*	−141	−2.66%	−63	−1.19%
f	1	0.02%	3	0.06%	131*	2.47%*	43*	0.81%*	130	2.45%	40	0.75%
fnc	106	2.00%	61	1.15%	7	0.13%	1	0.02%	−99	−1.87%	−60	−1.13%
fc	5	0.09%	2	0.04%	5*	0.09%*	0*	0%*	0	0%	−2	−0.04%
m	30	0.57%	0	0%	125*	2.36%*	21*	0.40%*	95*	1.79%*	21*	0.40%*
m0	5	0.09%	0	0%	0	0%	0	0%	−5	−0.09%	0	0%
X	2765	52.17%	2525	47.64%	3981	75.11%	3774	71.21%	1216	22.94%	1249	23.57%

* *p* < 0.05, Mann–Whitney *U*‐test.

**TABLE 4:** Number and percent of S (sound tooth surfaces), DNC (decayed non‐cavitated lesions), DC (decayed cavitated lesions), F (filled lesions), FNC (filled non‐cavitated lesions), FC (filled cavitated lesions), M (missing tooth surfaces, because of caries), M0 (missing tooth surfaces, because of other reasons than caries), X (not examined or excluded tooth surfaces) in year 2008, 2011, and caries increment between year 2008 and 2011 in asthmatic children and non‐asthmatic siblings (*n* = 106) in permanent teeth

	Year 2008				Year 2011				Caries increment over 3 years			
	Asthmatic children		Non‐asthmatic siblings		Asthmatic children		Non‐asthmatic siblings		Asthmatic children		Non‐asthmatic siblings	
Permanent teeth	Surfaces	%	Surfaces	%	Surfaces	%	Surfaces	%	Surfaces	%	Surfaces	%
S	3763	39.01%	3598	37.30%	5562	57.66%	5679	58.87%	1799	18.65%	2081	21.57%
DNC	22	0.23%	21	0.22%	97	1.01%	29	0.30%	75	0.78%	8	0.08%
DC	54*	0.56%*	11*	0.11%*	149*	1.54%*	12*	0.12%*	95*	0.98%*	1*	0.01%*
F	154	1.60%	57	0.59%	245*	2.54%*	74*	0.77%*	91*	0.94%*	17*	0.18%*
FNC	0	0%	0	0%	41*	0.43%*	1*	0.01%*	41*	0.43%*	1*	0.01%*
FC	0	0%	0	0%	21*	0.22%*	0*	0%*	21*	0.22%*	0*	0%*
M	0	0%	0	0%	14	0.15%	0	0%	14*	0.15%*	0*	0%*
M0	0	0%	20	0.21%	0	0%	20	0.21%	0	0%	0	0%
X	5653	58.60%	5939	61.57%	3517	36.46%	3831	39.72%	−2136	−22.14%	−2108	−21.85%

* *p* < 0.05, Mann–Whitney *U*‐test.

Asthmatic children had significantly (*p* < 0.05) more DC on their permanent teeth in year 2008 and 2011, and in year 2011 significantly more F, FNC, and FC (Table 4). There was a significantly higher increment over 3 years of DC, F, FNC, FC, as well as M on permanent teeth among asthmatic children.

Caries increment in our study was presented also according to Ismail et al. (2011)). In Tables 5 and 6, percent of dental caries transition between the baseline caries assessment in 2008 and the subsequent assessment after 3 years in 2011 in a tooth surface level in primary teeth among asthmatic children and their non‐asthmatic siblings is presented. In asthmatic children, progression from s in primary teeth to dc and f was present in significantly (*p* < 0.05) higher percent than in non‐asthmatic siblings. Also, transition from dnc to f and transition from dc to m were significantly more frequent in asthmatic children. Transition from dnc to s was more frequent in non‐asthmatic siblings, although not significantly.

**TABLE 5:** Percent of dental caries transition over 3 years at the tooth surface level in primary teeth among asthmatic children (*n* = 53)

Asthmatic children primary teeth	s	Dnc	Dc	f	Fnc	Fc	m	m0	x
s	46.00%	0.19%	1.40%*	1.68%*	0.23%	0.05%	1.12%	0%	49.35%
dnc	2.08%	4.17%	25.00%	37.50%*	0%	0%	8.33%	0%	22.92%
dc	1.04%	0%	3.13%	18.75%	0.52%	0.52%	34.90%*	0%	41.15%
f	2.83%	0%	2.83%	38.68%	0.94%	2.83%	0%	0%	51.89%
fnc	0%	0%	0%	0%	0%	0%	0%	0%	100%
fc	0%	0%	0%	0%	0%	0%	0%	0%	100%
m	0%	0%	0%	0%	0%	0%	100%	0%	0%
m0	0%	0%	0%	0%	0%	0%	0%	0%	100%
x	0%	0%	0%	0%	0%	0%	0%	0%	100%

* *p* < 0.05, Mann–Whitney *U*‐test.

**TABLE 6:** Percent of dental caries transition over 3 years at the tooth surface level in primary teeth among non‐asthmatic siblings (*n* = 53)

Non‐asthmatic siblings primary teeth	s	Dnc	Dc	f	Fnc	Fc	m	m0	x
s	54.38%	0.04%	0.04%*	0.42%*	0%	0%	0.27%	0%	44.86%
dnc	15.38%	53.85%	3.85%	3.85%*	0%	0%	3.85%	0%	19.23%
dc	0%	0%	4.41%	23.53%	0%	0%	19.12%*	0%	52.94%
f	0%	0%	0%	22.95%	1.64%	0%	0%	0%	75.41%
fnc	0%	0%	0%	0%	0%	0%	0%	0%	100%
fc	0%	0%	0%	50.00%	0%	0%	0%	0%	50.00%
m	0%	0%	0%	0%	0%	0%	0%	0%	0%
m0	0%	0%	0%	0%	0%	0%	0%	0%	0%
x	0.59%	0%	0%	0%	0%	0%	0%	0%	99.41%

* *p* < 0.05, Mann–Whitney *U*‐test.

Transition of dental caries over 3 years in permanent teeth is presented in Tables 7 and 8. In asthmatic children, a significantly higher percent of S in permanent teeth progressed to DC, F, and FC. Transition from F to FNC and FC were more frequent, as well as transition from X to DNC and DC, compared to caries transitions among non‐asthmatic siblings. Transition from DNC to S was more frequent in healthy siblings, although not significantly.

**TABLE 7:** Percent of dental caries transition over 3 years at the tooth surface level in permanent teeth among asthmatic children (*n* = 53)

Asthmatic children permanent teeth	S	DNC	DC	F	FNC	FC	M	M0	X
S	93.33%	1.49%	1.89%*	1.73%*	0.56%	0.37%*	0.32%	0%	0.32%
DNC	4.55%	36.36%	22.73%	31.82%	4.55%	0%	0%	0%	0%
DC	0%	1.85%	18.52%	72.22%	3.70%	0%	3.70%	0%	0%
F	0%	1.95%	3.25%	79.22%	11.04%*	4.55%*	0%	0%	0%
FNC	0%	0%	0%	0%	0%	0%	0%	0%	0%
FC	0%	0%	0%	0%	0%	0%	0%	0%	0%
M	0%	0%	0%	0%	0%	0%	0%	0%	0%
M0	0%	0%	0%	0%	0%	0%	0%	0%	0%
X	35.59%	0.51%*	1.03%*	0.87%	0%	0%	0%	0%	62.00%

* *p* < 0.05, Mann–Whitney *U*‐test.

**TABLE 8:** Percent of dental caries transition over 3 years at the tooth surface level in permanent teeth among non‐asthmatic siblings (*n* = 53)

Non‐asthmatic siblings permanent teeth	S	DNC	DC	F	FNC	FC	M	M0	X
S	98.69%	0.58%	0.03%*	0.36%*	0%	0%*	0%	0%	0.33%
DNC	38.10%	28.57%	23.81%	9.52%	0%	0%	0%	0%	0%
DC	0%	0%	36.36%	63.63%	0%	0%	0%	0%	0%
F	0%	0%	0%	98.25%	1.75%*	0%*	0%	0%	0%
FNC	0%	0%	0%	0%	0%	0%	0%	0%	0%
FC	0%	0%	0%	0%	0%	0%	0%	0%	0%
M	0%	0%	0%	0%	0%	0%	0%	0%	0%
M0	0%	0%	0%	0%	0%	0%	0%	100%	0%
X	35.63%	0.03%*	0.03%*	0%	0%	0%	0%	0%	64.30%

* *p* < 0.05, Mann–Whitney *U*‐test.

In the second part of the study, the analysis conducted on 446 asthmatic children showed that asthmatic children who used leucotriene antagonist had lower mean D_1_MFS (Table 9). Likewise, there was significant difference in caries experience between asthmatic children who were reported to have used higher (>150 μg) daily dose of inhaled glucocorticoid and those who were reported to use lower (≤150 μg) dose, higher doses were associated with higher mean D_12_MFS. Also, daily consumption of milk and cheese was associated with lower mean D_12_MFS. There was no significant difference between the groups in terms of mean child's age.

**TABLE 9:** Influence of various confounding factors on mean D_1_MFS and D_12_MFS in 446 asthmatic children

Factors	*n*	D_1_MFS (mean ± SD)	D_12_MFS (mean ± SD)
Medication—Daily glucocorticoid dose			
≤150 μg	113	–	6.20* ± 8.90
>150 μg	333	–	8.33* ± 10.11
Medication—Leucotriene antagonist use			
No	363	5.55* ± 7.74	–
Yes	83	3.43* ± 5.15	–
Eating habits—Daily consumption of milk and cheese			
No	333	–	8.15* ± 8.95
Yes	113	–	6.60* ± 9.39

* *p* < 0.05, Mann–Whitney *U*‐test.

## 4 DISCUSSION

A link between asthma and dental caries has long been discussed (Alavaikko et al., 2011), but the results seem to be conflicting, thus the present study investigated the influence of childhood asthma and exposure to anti‐asthmatic medications on dental caries development among children with asthma. Various stages of caries process and their longitudinal development were recorded. Furthermore, caries risk factors among asthmatic children were examined. The study demonstrated that asthmatic children had significantly lower prevalence of sound tooth surfaces than their non‐asthmatic siblings, and significantly a greater number of non‐asthmatic siblings were caries‐free when compared with the asthmatic children (Table 1). Furthermore, asthmatic children had significantly higher mean d_12_fs compared to non‐asthmatic siblings on their primary teeth, as well as significantly higher mean D_12_MFS on their permanent teeth (Table 2), and there was a higher increment of D_12_MFS scores over 3 years (Figure 1). Thus our prospective results reconfirmed and strengthened conclusions from previous that asthma is associated with dental caries (Agostini et al., 2019; Alavaikko et al., 2011). However, alternative research has failed to demonstrate an association between dental caries and asthma (Flexeder et al., 2020; Maupomé et al., 2010). It is important to note that the present study, unlike previous studies, recorded non‐cavitated carious lesions. Inclusion of early stages of the caries process improves the precision of studies (Ismail et al., 2007). This was the case for only two other cohort studies (Flexeder et al., 2020; Stensson, Wendt, Koch, Nilsson, et al., 2010), that examined the association between asthma and dental caries.

The study further demonstrated the importance of caries progression based on longitudinal data in contrast to using only a cross‐sectional assessment. A greater number of the primary tooth surfaces recorded as sound in asthmatic children in 2008 were now observed to have progressed to decayed non‐cavitated (dnc) or decayed cavitated (dc) surfaces within 3 years (Tables 5 and 6), thus significantly less sound tooth surfaces were observed in asthmatic children when compared with their non‐asthmatic siblings. Likewise, a large proportion of baseline dnc progressed to a more severe caries, (dc lesions) in asthmatic children, while in their non‐asthmatic siblings, majority of baseline dnc remained non‐cavitated. Similarly, in asthmatic children, significantly more of baseline dc progressed to missing (m) surfaces and to tooth loss as a result of caries, unlike their non‐asthmatic siblings. There was a significantly higher (4.5‐fold) increment over 3 years of m on primary teeth (Table 3) among asthmatic children. These results are in agreement with the report of Kankaala et al. (1998) in which the asthma patients had more extractions of primary molars owing to caries than the non‐asthmatic controls. Furthermore, in the present study, a significantly higher percentage of baseline dnc and dc lesions were now treated with fillings in asthmatic children than their non‐asthmatic siblings. Similarly, in permanent teeth of asthmatic children, less sound surfaces in 2008 remained sound in 2011, significantly greater number progressed to DNC or DC within 3 years compared to non‐asthmatic siblings (Tables 7 and 8). Unsurprisingly, primary teeth were more susceptible to caries development than permanent teeth, the number of primary sound surfaces that remained sound during this 3 years were two times smaller, and this is in agreement with previous studies that demonstrated children in younger age groups to be more susceptible to caries than older children (Eggertsson & Ferreira‐Zandona, 2009; McDerra et al., 1998). During the same period, a significantly higher percent of baseline DNC and DC lesions in asthmatic children were treated with fillings. A significantly higher increment of DC, F, FNC, FC, as well as M was present among asthmatic children (Table 4). By and large, higher increment of filled teeth surfaces in both dentitions among asthmatic children is an indication of a higher treatment need among this group. This observation is consistent with the report of Kankaala et al. (1998) that asthmatic children receiving inhaled glucocorticoids showed higher filling increments in the primary molars.

Contrary to previous studies where healthy controls were not siblings and children with asthma showed higher intake of sugary drinks (Stensson, Wendt, Koch, Nilsson, et al., 2010; Stensson, Wendt, Koch, Oldaeus, et al., 2010), in the present study, asthmatic children did not differ significantly from their non‐asthmatic siblings with respect to time since last dental visit, parents' education, oral hygiene, dietary habits, fluoride intake, frequency of drinking sweet drinks. The discrepancies between conclusions of different studies can be partly explained by different study populations. Asthma is a heterogeneous disease (Reddel et al., 2015) with different severities and can have clinical seasonal fluctuations. In the studies of Kankaala et al. (1998) and Ersin et al. (2006), they included children that had been taking medications, McDerra et al. (1998) and Reddy et al. (2003) included children currently using β2‐agonists, whereas Milano et al. (2006) those that have used β2‐agonists for at least 1 year. On the other hand, Stensson, Wendt, Koch, Oldaeus, et al. (2010) included adolescents with only severe and very severe asthma with daily glucocorticoid treatment for a minimum of 4 years. In the study of Shulman et al. (2001), no significant associations between asthma and higher prevalence of dental caries were found. In this study, less than 45% of the children with severe asthma were on prescribed anti‐asthmatic medication. Only a few children from the moderate and severe asthma group were using glucocorticoids, and only for a short duration. Flexeder et al. (2020) also found no significant association between caries and asthma; however, in their study, information on the frequency of metered‐dose inhalers medication was not available. Moreover, there was no clinical ascertainment of the parentally reported diagnosis. In contrast, in the present study, asthmatic medications were taken daily for at least 1 year; 10.1% of asthmatic children included in 2008 took medications for 1 year, 71.1% for 2–8 years, and 18.8% for 9–17 years.

In asthmatic children significantly lower caries experience was found in children with lower daily dose of glucocorticoid and in those who used leucotriene antagonist (Table 9). Children used glucocorticoid daily and bronchodilatator as circumstances required, making it impossible to study the impact of glucocorticoid or bronchodilatator alone. The results are consistent with previous studies that reported association between increased caries experience and increased frequency and duration of asthma medication use (Chellaih et al., 2016; Chumpitaz‐Cerrate et al., 2020; Milano et al., 2006; Rezende et al., 2019) or increased severity of asthma (Reddy et al., 2003). Previous studies also reported an inverse relationship between asthma severity and salivary flow rate (Paganini et al., 2011), a negative correlation between the duration of medication and salivary pH, and a positive correlation between duration of illness and the salivary levels of Streptococcus mutans in the asthmatics (Botelho et al., 2011; Ersin et al., 2006), which are well‐known risk factors for increased caries experience (Selwitz et al., 2007). Thus the increased caries prevalence, severity, and progression observed among the asthmatic children in the present study could be attributed to low salivary flow rate, low salivary pH, and high levels of Streptococcus mutans in the asthmatics as demonstrated by previous studies (Botelho et al., 2011; Ersin et al., 2006; Paganini et al., 2011). It is not surprising to have low salivary flow rate among the asthmatic children due to the use of β2‐agonists, which are known to affect the salivary gland functions. Thus the asthmatic children lack the protective actions of saliva against dental caries, which includes but not limited to buffering of bacterial acid and antibacterial effect, hence the low pH and high Streptococcus mutans levels. Besides, some of the inhaled metered medications contain lactose, a carbohydrate that is fermentable by cariogenic bacteria to produce acids that causes tooth demineralization. The effect of this would obviously be worse with the limited amount of saliva and high of Streptococcus mutans. Of course, it is well established that frequent sugar intake results to high proliferation of Streptococcus mutans. Also frequent intake and prolonged presence of sugar on tooth surfaces result to constant acid production by bacteria, and hence caries manifestation. Furthermore, as inhaled β2‐agonists increase the surface roughness of restorative materials (Ayaz et al., 2014), higher number of filled surfaces may lead to more plaque retention and bacterial adherence, and thus to the observed higher caries susceptibility. The results showed that additional leucotriene antagonist use was a protective factor, a likely explanation is that increasing the dose of inhaled glucocorticoids to control symptoms was avoided (Castillo et al., 2017).

In asthmatic children, lower mean D_12_MFS was associated with daily consumption of milk and cheese (Table 9). This finding is in agreement with the available literature, milk products, if unsweetened by added sugars, may have some caries protective potential (Woodward & Rugg‐Gunn, 2020). They have the ability to buffer organic acids formed by sugar fermentation, adsorption of milk proteins onto the surface of the teeth protects them against demineralization, and they enhance remineralization (Kahama et al., 1998). Greater milk and cheese intake could also be an indicator of other healthy dietary behaviors. This is in line with the observations of previous studies (Samec et al., 2013; Selwitz et al., 2007), showing that behavioral, and lifestyle‐related factors are important.

Biologically plausible regression, that is, transition from decayed non‐cavitated lesions to sound in permanent as well as in primary teeth was observed but more in non‐asthmatic children than their asthmatic counterparts. It is known that caries process may reverse, mainly while the surface of the lesion is still intact, with increased deposition of minerals in the lesion area and reduction in pore size of the arrested lesion (Eggertsson & Ferreira‐Zandona, 2009). The transition being more frequent in non‐asthmatic siblings suggests that there are factors that tilt the biochemistry of the oral environment of asthmatic children toward high caries risk, indicating that asthmatic children are at a high caries risk status, unlike their non‐asthmatic siblings.

In conclusion, the present study has shown that children with asthma who had used anti‐asthmatic medicines had higher caries experience in primary and permanent teeth. For the first time, patterns of caries development were presented, and higher caries progression over time shown. The study has identified various factors associated with caries experience, including anti‐asthmatic medicines. These findings will facilitate the development of a preventive program for this high‐caries‐risk group of children.

Considering that these children with asthma are at high caries risk, we recommend extra caries preventive measures, in addition to low sugary diet and brushing teeth at least twice daily with fluoridated toothpaste. Such extra measures as rinsing mouth with water immediately following medicine administration and using products that increases the bioavailable calcium and phosphate in the oral environment such as toothpaste/or gel containing amorphous calcium phosphate or hydroxyapatite (Enax et al., 2019; Meyer & Enax, 2018). Regular visits to the dentist should be mandatory for these children. In‐office quarterly application of fluoride varnish or 6 monthly application of silver diammine fluoride can be implemented, and if necessary microinvasive and invasive treatment (Martignon et al., 2019; Slayton et al., 2018).

## CONFLICT OF INTEREST

The authors declare no conflict of interest.

## AUTHOR CONTRIBUTIONS

All authors have made substantial contributions to conception and design of the study. Tomi Samec and Janja Jan collected and analyzed the data. Tomi Samec, Janja Jan, and Bennett Tochukwu Amaechi led the writing.

## ETHICS STATEMENT

The study protocol was approved by ethics committee at the Ministry of Health in Slovenia (No. 165/07/09). All the parents gave written informed consent for inclusion in this study.

## Data Availability

The data that support the findings of this study are available on request from the corresponding author. The data are not publicly available due to privacy or ethical restrictions.
